# Short-Term Effects of Chewing on Task Performance and Task-Induced Mydriasis: Trigeminal Influence on the Arousal Systems

**DOI:** 10.3389/fnana.2017.00068

**Published:** 2017-08-08

**Authors:** Maria Paola Tramonti Fantozzi, Vincenzo De Cicco, Massimo Barresi, Enrico Cataldo, Ugo Faraguna, Luca Bruschini, Diego Manzoni

**Affiliations:** ^1^Department of Translational Research and of New Surgical and Medical Technologies, University of Pisa Pisa, Italy; ^2^Institut des Maladies Neurodégénératives, University of Bordeaux Bordeaux, France; ^3^Department of Physics, University of Pisa Pisa, Italy; ^4^Department of Developmental Neuroscience, IRCCS Foundation Stella Maris Pisa, Italy; ^5^Department of Surgical, Medical, Molecular Pathology and Critical Care Medicine, University of Pisa Pisa, Italy

**Keywords:** chewing, trigeminal input, locus coeruleus, mydriasis, cognitive performance

## Abstract

Trigeminal input to the ascending activating system is important for the maintenance of arousal and may affect the discharge of the noradrenergic neurons of the *locus coeruleus* (LC), whose activity influences both vigilance state and pupil size, inducing mydriasis. For this reason, pupil size evaluation is now considered an indicator of LC activity. Since mastication activates trigeminal afferent neurons, the aims of the present study, conducted on healthy adult participants, were to investigate whether chewing a bolus of different hardness may: (1) differentially affect the performance on a cognitive task (consisting in the retrieval of specific target numbers within numerical matrices) and (2) increase the dilatation of the pupil (mydriasis) induced by a haptic task, suggesting a change in LC activation. Results show that chewing significantly increased both the velocity of number retrieval (without affecting the number of errors) and the mydriasis associated with the haptic task, whereas simple task repetition did not modify either retrieval or mydriasis. Handgrip exercise, instead, significantly decreased both parameters. Effects were significantly stronger and longer lasting when subjects chewed hard pellets. Finally, chewing-induced improvements in performance and changes in mydriasis were positively correlated, which suggests that trigeminal signals enhanced by chewing may boost the cognitive performance by increasing LC activity.

## Introduction

Earlier experiments have shown that trigeminal signals play a particularly important role in the control of cortical desynchronization and arousal (Roger et al., 1956). This can be accounted for by the influence exerted by trigeminal afferents on the Ascending Reticular Activating System (ARAS). Trigeminal primary and/or secondary afferents reach the pontomedullary reticular formation (McKinley and Magoun, 1942), the noradrenergic neurons of the locus coeruleus (LC) (Cedarbaum and Aghajanian, 1978; Luo et al., 1991; Craig, 1992; Couto et al., 2006), the cholinergic neurons located within the pedunculopontine and the laterodorsal tegmental nucleus (Semba and Fibiger, 1992), the tuberomammillary hypothalamic histaminergic neurons (Fujise et al., 1998; Sakata et al., 2003) and the thalamic non-specific intralaminar and midline nuclei (Krout et al., 2002; Clascá et al., 2012). Although it has been claimed that mesencephalic trigeminal neurons (see Wang and May, 2008) are electrotonically coupled to LC neurons (Matsuo et al., 2015), there is at present no evidence of this statement, beyond a Fluorogold labeling passage from mesencephalic trigeminal neurons to the LC area (Fujita et al., 2012), which only implies the possibility of such a speculation. LC neurons modulate arousal (Samuels and Szabadi, 2008; Carter et al., 2010), sensorimotor excitability (Matsutani et al., 2000) and provide an essential contribution to arousal-associated (Bradshaw, 1967) pupil mydriasis (Gabay et al., 2011), which reflects “mental efforts” (Hess and Polt, 1964) and task performance (Rajkowski et al., 1993). In fact, LC controls the preganglionic parasympathetic neurons of the Edinger–Westphal nucleus (Breen et al., 1983), which innervate the iris constrictor, inhibiting their discharge through an α2-mediated mechanism (Szabadi and Bradshaw, 1996; Samuels and Szabadi, 2008). Such inhibition is necessary to increase the pupil size, since the tonic activity of the iris constrictor would prevent pupil enlargement by dilatator iris (Wilhelm et al., 2011). As a consequence, the LC neurons activity is strongly and positively correlated with the pupil size, both in animals (Rajkowski et al., 1993, 1994; Joshi et al., 2016) and humans (Alnaes et al., 2014; Murphy et al., 2014). For this reason, several studies have utilized the pupil size as an index of LC activity (Silvetti et al., 2013; Hoffing and Seitz, 2015; Kihara et al., 2015; Hayes and Petrov, 2016; see also Laeng et al., 2013). The connection of the trigeminal system with the ARAS and LC suggests that the modifications of trigeminal input occurring during chewing may induce relevant changes in the whole brain, leading to an enhancement in the arousal/alertness level and, as a consequence, in the cognitive performance (Sakamoto et al., 2009).

Mastication is driven by a central pattern generator (Dellow and Lund, 1971), controlled by oral sensory feedback (Appenteng et al., 1982; Lund, 1991), according to the changes in the consistency and texture of the food bolus (Horio and Kawamura, 1989). Masticatory muscle activation (Peyron et al., 2002), together with feedback signals from periodontal receptors and muscle spindles (Lavigne et al., 1987), increases with food hardness and influences brain function. In fact, animals submitted to long-term soft-diet feeding undergo a decrease in learning and memory (Tsutsui et al., 2007; Weijenberg et al., 2013), whereas mastication prevents the degradation of brain functions (Gatz et al., 2006; Okamoto et al., 2010; Ohkubo et al., 2013). Moreover, animal models showed that bilateral molar extractions, leading to long-term masticatory dysfunction, decrease the number of pyramidal cells in the hippocampal CA1 and gyrus dentatus (Oue et al., 2013), with impairment of spatial learning and memory in water maze tests (Kato et al., 1997). These deficits increase with aging and time after teeth loss (Onozuka et al., 2000). On top, teeth loss increases the proliferation and the hypertrophy of the astrocytes within the hippocampus, as it occurs following neuronal degeneration and senescence processes (Onozuka et al., 2000), while decreasing c-Fos expression during spatial task (Watanabe et al., 2002), dendritic spines density (Kubo et al., 2005) and neurogenesis (Aoki et al., 2010).

In humans, beyond the trophic, long-term effects that chewing exerts on the brain (Gatz et al., 2006; Okamoto et al., 2010), gum chewing improves cognitive processing speed (Hirano et al., 2013), alertness (Allen and Smith, 2012; Johnson et al., 2012), attention (Tucha et al., 2004), memory and learning (Allen et al., 2008; Smith, 2009); it also reduces reaction times (Hirano and Onozuka, 2014) and event-related potentials latencies in an auditory oddball paradigm (Sakamoto et al., 2009). Moreover, shortening of the visual reaction time in a button press task following chewing (Hirano and Onozuka, 2014) was found associated with an increase of the blood-oxygen-level dependent signal in the anterior cingulate, left frontal gyrus and motor related regions. On the other hand, chewing-induced improvement of short-term memory processing was coupled to an increased blood flow in the middle frontal gyrus of the dorsolateral prefrontal cortex, the right premotor cortex, precuneus, thalamus, hippocampus and inferior parietal lobe (Hirano et al., 2008).

Chewing-induced advantages in cognitive performance were observed following, but not during chewing bouts for a time period of 15–20 min (Onyper et al., 2011). The consistency of the chewed gum pellet influences chewing-induced performance changes. In fact, at variance with an ordinary chewing gum, a soft chewing gum like bubble gum did not improve memory (Davidson, 2011). On the other hand, the prolonged chewing of a hard gum significantly increased the fatigue of the masticatory muscles, blunting cognitive performance (Farella et al., 2001).

Chewing effects on cognitive performance and arousal could be related to trigeminal action on different ARAS components. More specifically, the involvement of the LC should result in an increased task-related mydriasis, due to the coupling between LC activity and pupil dilatation, well documented by several investigations (Rajkowski et al., 1993, 1994; Alnaes et al., 2014; Murphy et al., 2014; Joshi et al., 2016). Therefore, based on the assumption of the proposed LC contribution to chewing effects on the brain, a correlation can be expected between chewing-induced changes in performance and in task-related mydriasis. Thus, the aim of the present study was to investigate whether short bouts of masticatory chewing influence (1) the velocity of retrieval of specific target numbers within numerical matrices and relative errors as well as (2) the mydriasis induced by a haptic task. Finally, we tested whether chewing induced changes in cognitive performance and mydriasis are correlated with each other.

## Materials and Methods

### Subjects

This study was carried out in accordance with the recommendations of the Ethical Committee of the Pisa University. According to the Declaration of Helsinki, each subject signed an informed consent, approved by the local Ethical Committee. Experiments were performed in 30 right-handed subjects (15 females) aged between 18 and 55 years (36.3 ± 12.5), not affected by pain in the masticatory/neck muscles and by neurological, psychiatric, metabolic or endocrine diseases.

### Experimental Procedure

Subjects were asked to avoid caffeine and smoking for at least 2 h before the experimental session. Each subject underwent 4 experimental sessions (no activity, handgrip, soft pellet, hard pellet), separated by at least 24 h within 4–6 days. In each session subjects were engaged in activities lasting 2 min (soft pellet, hard pellet, handgrip) or invited to relax for 2 min, without specific instructions (no activity). Activities consisted of chewing a custom-made hard pellet, chewing a commercially available soft gum pellet, and rhythmically squeezing an anti-stress ball of 6 cm in diameter, respectively. Each subject performed all motor activities according to his/her own preferred rate, on the preferred side during the first minute and on the other side for the remaining time. When changing the side, the soft (but not the hard) pellet was discarded and a new pellet was delivered to the subject. The session order was pseudo-randomly varied among participants. In each session subjects were studied at T0 (control), T7 and T37, i.e., 5 min before the beginning, soon after and 30 min following the end of the activity/no activity period, respectively. Thus, the activity/no activity period began, in each session, 5 min after T0 and ended just before T7. Between T7 and T37 measurements, subjects were invited to relax.

### Variables

All the listed variables were studied at T0, T7 and T37.

**FIGURE 1:**
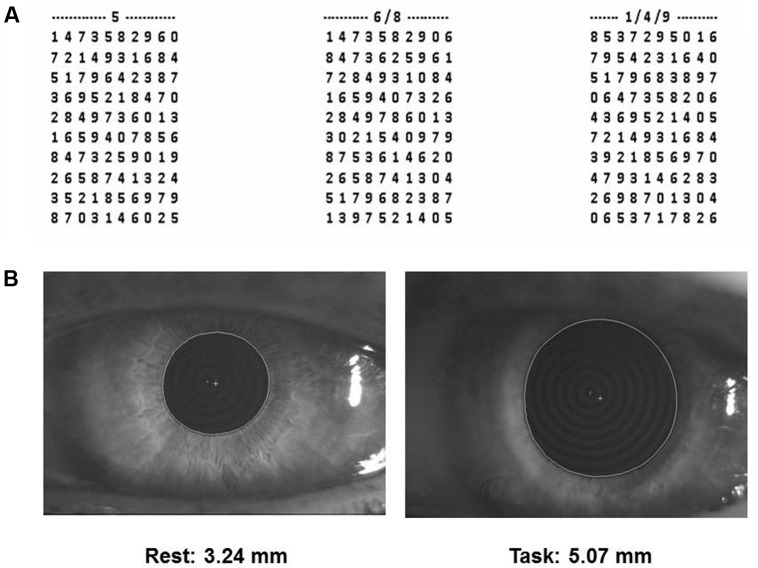
Spinnler-Tognoni matrices and pupil size recordings. **(A)** Spinnler-Tognoni matrices. The test consists in identifying the target numbers indicated above each matrix. **(B)** Example of pupillometric recording performed at rest (left side) and during Tangram execution (right side).

(1) Performance Index, Scanning Velocity and Error Rate in a cognitive task based on a modified version of the Spinnler-Tognoni numeric matrices test (Spinnler and Tognoni, 1987) (**Figure 1A**).

In this task, the subject seated in front of a table, where the experimenter displayed a paper sheet containing three numerical matrices of 10 lines and 10 columns. The subjects had been previously instructed to underline with a pencil the number 5 in the first matrix from the left, the numbers 6 and 8 in the second and the numbers 1, 4 and 9 in the third (**Figure 1A**). They were invited to retrieve as many target numbers as possible, scanning sequentially each matrix line. The target numbers were 60 out of 300 included within the 3 matrices. The numbers retrieved in 15 s were counted and divided by 15, thus obtaining the velocity of number retrieval, indicated as Performance Index. Moreover, we evaluated the sum of missed target numbers and non-target numbers wrongly underlined, which was divided by 15 for obtaining the Error Rate. Finally, we counted all the numbers (target and non-target) scanned and obtained the Scanning Velocity, by dividing it by 15.

The matrices presented at T0, T7 and T37 differed for the position of the target numbers, thus subjects could not benefit of previous spatial information for speeding up their performance.

(2) Pupil size at rest and during execution of a haptic task (**Figure 1B**) based on Tangram, a puzzle of triangular, square and parallelogram-shaped forms. During both rest and task measurements the subjects were seated with the head within a pupillometer. In the first case, the subjects were invited to relax. Measurements on both sides were taken. During task measurements, a piece of the puzzle (the parallelogram) was removed by the experimenter and placed in the right hand of the subjects, who had to reposition it in its original place without looking at their hand and the pupil size was monitored as soon as they began to fit the piece in its proper place. Left and right pupil measurements were collected in separate task repetitions. All the subjects performed this task for training, before the beginning of the first experimental session.

Pupil size measurements (mm) were performed in standard conditions of artificial lighting by using a corneal topographer-pupillographer (MOD i02, with chin support, CSO, Florence, Italy) made up of a standard illuminator (halogen lamp, white light), a camera sensor CCD1/3”, with a 56 mm working distance. The operator monitored the iris image (**Figure 1B**) by the camera (acquisition time 33 ms). Measurements were performed in photopic conditions (40 lux) and values were displayed online on the computer screen. During pupil size evaluation (both at rest and during task) the dental arches were not in contact.

Pupillographic measurements have been previously used to indirectly track the activity of LC neurons (Silvetti et al., 2013; Hoffing and Seitz, 2015; Kihara et al., 2015; Hayes and Petrov, 2016; see also Laeng et al., 2013; De Cicco et al., 2014, 2016).

### Pellets and Anti-stress Ball

The anti-stress ball squeezed by the subjects during the handgrip task (TB600 Artengo, Italy) was made by a polyurethane foam, characterized by a constant hardness (defined as the material’s resistance to indentation when a static load is applied) of 30 Shore OO, where 0 and 100 Shore OO correspond to maximal and no indentation, respectively. The soft pellet consisted of a commercially available chewing gum (Vigorsol, Perfetti, Italy), with an initial hardness of about 20 Shore OO. It was white in color, made of base gum with the main taste of liquorice (2.0 cm × 1.0 cm × 0.5 cm in size). The hard pellet was instead manufactured (OCM Projects, Italy) by using a silicon rubber (gls50, Prochima, Italy) and was characterized by a reticular structure. The material had a constant hardness (60 Shore OO), unmodified during chewing. The induced deformation was approximately proportional to the muscular force applied (spring constant = 15.7 N/m) and the material quickly recovers its original shape when the pressure ended. The pellet was cylindrical in shape (1.0 cm × 1.0 cm × 1.5 cm). It was gray in color, sugar free, odor and tasteless. The pellets were self-administered and the subject had visual access to the pellet right before the administration.

### Statistical Analysis (SPSS.13)

The average pupil size (left/right) at rest and during the haptic task, their difference (i.e., the task-induced mydriasis), the Performance Index, the Error Rate and the Scanning Velocity were analyzed. Correlations between variables were assessed by linear regression analysis. The effects of the different motor activities on the variables listed above at the different times were analyzed by a 4 Conditions (no activity, handgrip, soft pellet, hard pellet) × 3 Times (T0, T7, T37) repeated measures ANOVA, with Gender as a between-subjects factor. Since several variables showed a correlation with age, the latter was used as a covariate. The Greenhouse-Geisser ε correction was used when requested. Significance was set at *P* < 0.05. *Post hoc* comparisons were performed by paired *t*-test. In addition, the differences between T7 and T37 values with respect to T0 (ΔT7 and ΔT37, respectively) were computed for each condition and compared by paired *t*-test.

## Results

### Correlation of Performance and Pupil Size with Age

The average values of Performance Index and Scanning Velocity recorded in control condition (T0) were negatively correlated with age (Performance Index: *r* = 0.491, *P* < 0.006, *Y* = -0.02*X*+2.48; Scanning Velocity: *r* = 0.588, *P* < 0.001, *Y* = -0.096*X*+16.435). Similar trends with comparable slope values were observed for both males and females, although, in the latter population, the correlation between Performance Index and age did not reach the significance level. Average Error Rate at T0 was not significantly correlated with age.

The T0 values of pupil size at rest and during the haptic task were highly correlated with each other (*r* = 0.932, *P* < 0.0005, *Y* = 1.131*X*+0.901), without differences between males and females and both of them exhibited significant negative correlation with age. Similar regression lines were observed for values obtained at rest (*r* = 0.648, *P* < 0.0005, *Y* = -0.039*X*+5.268) and during the haptic task (*r* = 0.597, *P* < 0.001, *Y* = -0.044*X*+6.875), independently of gender. The mydriasis observed during Tangram performance did not correlate with age neither in males nor in females.

### Influence of Motor Activity on Spinnler-Tognoni Matrices Processing

**FIGURE 2:**
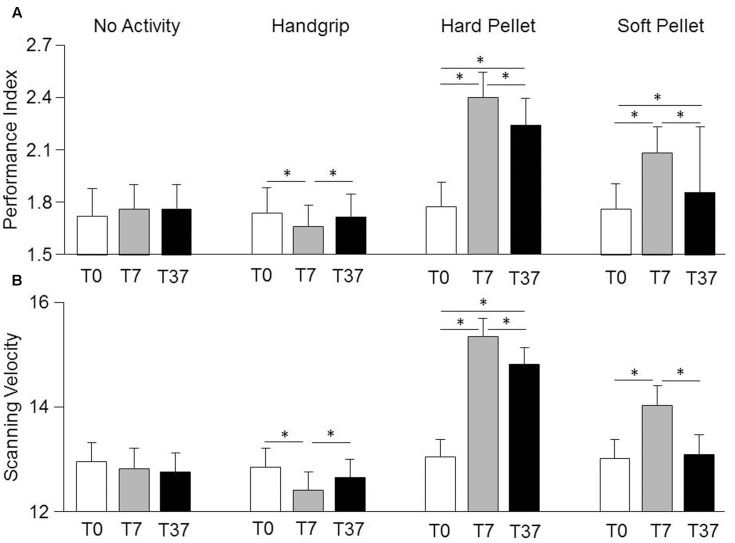
Changes in Performance Index and Scanning Velocity induced by various sensorimotor activities. Columns represent the mean values of the Performance Index **(A)** and of the Scanning Velocity **(B)** observed in the 4 conditions (no activity, handgrip, hard pellet, soft pellet) and at the different times tested (T0, T7, T37) are represented by the height of the corresponding columns. In **(A,B)**, the error bars represent Standard Errors. Asterisks refer to significant differences between T0, T7 and T37, as indicated by the horizontal lines. See Supplementary Table 2 for details.

Relevant effects and interactions of Condition, Time, Gender on Spinnler-Tognoni matrices processing are detailed in the Supplementary Table 1. In particular, significant Condition × Time interactions were observed for Scanning Velocity [*F*(6,162) = 3.76, *P* < 0.002] and Performance Index [*F*(6,162) = 9.48, *P* < 0.0005], whose decomposition can be found in the Supplementary Table 2, together with data relative to the actual number of retrieved and missed target items (Supplementary Table 3). Both variables were not significantly modified by simple test repetition neither at T7 nor at T37 with respect to T0 (**Figure 2**). They decreased significantly soon after the handgrip (T7) but not at T37 with respect to T0 (**Figure 2**). Chewing hard and soft pellet significantly increased the Scanning Velocity and Performance Index at T7. When chewing hard pellet both variables were still significantly enhanced at T37, whereas, at this time, only the Performance Index was still larger than in control condition (**Figure 2**). Analysis of ΔT7 and ΔT37 showed that the differences in Performance Index and Scanning Velocity at T7 and T37 with respect to T0 induced by chewing hard pellet were always significantly larger than those obtained by chewing soft pellet (ΔT7, *P* < 0.0005; ΔT37, *P* < 0.0005).

No Gender effects were observed. However, due to the low values of the η^2^ coefficients for the Condition × Time × Gender interaction (Performance Index = 0.038, Scanning Velocity = 0.029) significant gender differences could have been present. In fact, in the handgrip condition the drop in Scanning Velocity and Performance Index observed at T7 with respect to control was observed in females (Performance Index: from 1.90 ± 0.66, SD, numb/s to 1.74 ± 0.58, *P* < 0.001; Scanning Velocity: from 13.56 ± 2.56 to 12.84 ± 2.32, SD, numb/s, *P* < 0.002), but not in males (Performance Index: from 1.56 ± 0.32 to 1.58 ± 0.30, NS; Scanning Velocity: from 12.12 ± 1.46 to 12.00 ± 1.34, NS). At T37 no significant differences with respect to control were found in both females (Performance Index: 1.84 ± 0.62; Scanning Velocity: 13.30 ± 2.34) and males (Performance Index: 1.60 ± 0.28; Scanning Velocity: 12.00 ± 1.22). In the no activity, hard pellet and soft pellet conditions, males and females showed a similar behavior.

**FIGURE 3:**
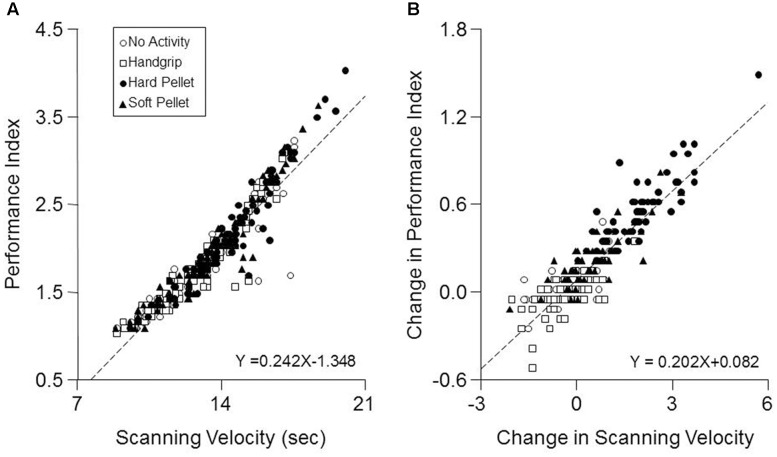
Correlation between Performance Index and Scanning Velocity. **(A)** The values of Performance Index obtained in the different conditions and times investigated have been plotted as a function of the corresponding Scanning Velocity values. **(B)** Changes in Performance Index obtained at T7 and T37 with respect to control values (T0) have been plotted as a function of the corresponding changes in Scanning Velocity. In **(A,B)** the dashed lines are regression lines of all the data points. Open circles: no activity condition. Open squares: handgrip condition. Dots: hard pellet condition. Filled triangles: soft pellet condition.

Only a significant Time effect was observed for the Error Rate, which decreased significantly from 0.28 ± 0.20 (T0) to 0.22 ± 0.14 (T7, *P* < 0.0005) and to 0.22 ± 0.18 (T37, *P* < 0.0005) independently from the performed activity. As shown in **Figure 3A** when data obtained at all times and conditions were pooled together, a significant correlation was found between the Performance Index and the Scanning Velocity (*r* = 0.94, *P* < 0.0005, *Y* = 0.242*X*-1.348). This relation was confirmed in each condition separately. On the other hand, increasing the Scanning Velocity did not significantly modify the Error Rate. Moreover, as shown in **Figure 3B**, also the pooled changes in Performance Index and Scanning Velocity obtained at T7 and T37 with respect to T0 correlated between each other (*r* = 0.902, *P* < 0.005, *Y* = 0.202*X*+0.082).

Independently from the Scanning Velocity, the performance accuracy indicated by the Performance Index was near maximal for all activities and times, as revealed by the saturation of percentage of retrieved targets, ranging from 99.2 ± 2.03 to 98.0 ± 2.66, for the different combinations of times and conditions.

### Influence of Motor Activity on Pupil Size at Rest and during Task Execution

**FIGURE 4:**
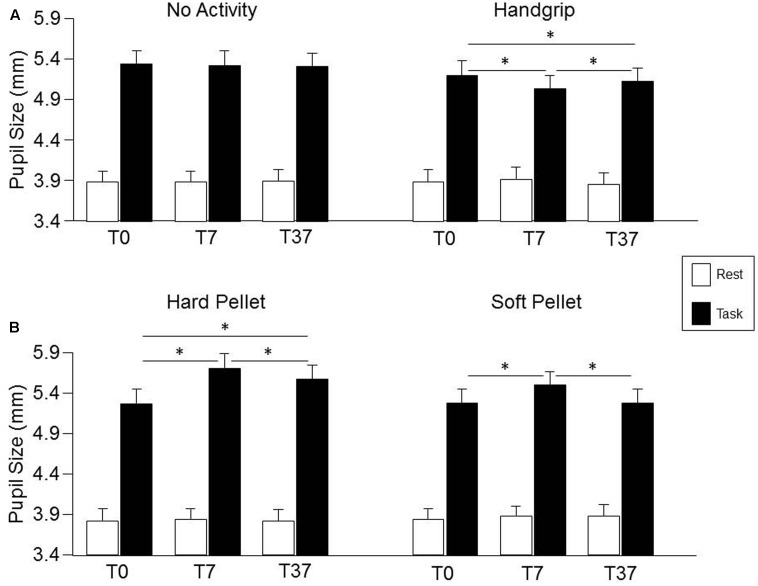
Changes in pupil size induced by the different sensorimotor activities. Columns represent the mean values of pupil size at rest (open columns) and during task (black columns) in the “no activity” and “handgrip” conditions **(A)** and in the “hard pellet” and “soft pellet” conditions **(B)** at T0, T7, T37. In **(A,B)**, the error bars represent Standard Errors. Asterisks refer to significant differences between T0, T7 and T37, as indicated by the horizontal lines. See Supplementary Table 5 for details.

Condition, Time, Gender effects and their interactions on pupil size are detailed in the Supplementary Table 4. No significant effects were observed for pupil size at rest, while a significant Condition × Time interaction was observed for pupil size during the haptic task [*F*(6,162) = 8.63, *P* < 0.0005], as well as for task related mydriasis [*F*(6,162) = 7.14, *P* < 0.0005], whose decomposition can be found in the Supplementary Table 5. As shown in **Figure 4** (lower row), with respect to control (T0), pupil size during haptic task significantly increased at both T7 and T37 after chewing hard pellet, while only at T7 after chewing soft pellet (**Figure 4**, lower row). On the other hand, it decreased at both T7 and T37 after handgrip performance (**Figure 4**, upper row). No change was induced at any time by simple task repetitions (**Figure 4**, upper row). Similar results were obtained for task related mydriasis, which can be appreciated in **Figure 4** by comparing data obtained at rest and during the task (Supplementary Table 5). Task-related mydriasis exhibited also a significant Gender effect sustained by higher values in males (1.59 ± 0.40, SD, mm) with respect to females (1.34 ± 0.40, SD, mm), independently from times and conditions.

The changes in pupil size (during haptic task) and in task-related mydriasis observed after chewing hard pellets were significantly larger than those observed after chewing soft pellets, both at T7 (*P* < 0.0005 for both parameters) and at T37 (*P* < 0.0005 for both parameters).

**FIGURE 5:**
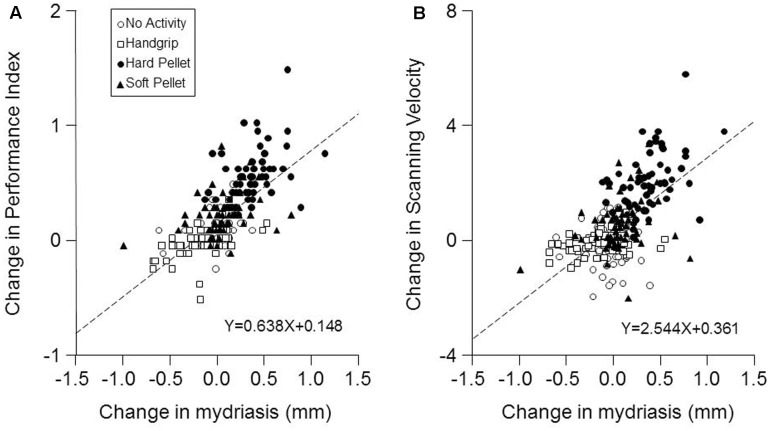
Relation between the changes in Performance Index and Scanning Velocity with respect to control (T0) induced by the different conditions and the corresponding changes in mydriasis. The changes in Performance Index **(A)** and in the Scanning Velocity **(B)** at T7 and T37 with respect to T0 (control) have been plotted as a function of the corresponding changes in task-induced mydriasis. In **(A,B)** the dashed lines are regression lines of all the data points. Open circles: no activity condition. Open squares: handgrip condition. Dots: hard pellet condition. Filled triangles: soft pellet condition.

Finally, as shown in **Figure 5**, a strong correlation was found between the changes in Performance Index and in Scanning Velocity induced by the various activities (at both T7 and T37) and the corresponding changes in mydriasis (Performance Index: *r* = 0.688, *P* < 0.0005, *Y* = 0.638*X*+0.148; Scanning Velocity: *r* = 0.615, *P* < 0.0005, *Y* = 2.544*X*+0.361). These correlations were observed in the handgrip, hard pellet and soft pellet conditions. In the no activity condition only changes in Performance Index were correlated with those in mydriasis.

## Discussion

The present findings indicate that short bouts of masticatory activity improve the velocity of number retrieval and matrix scanning and increase the task-induced mydriasis, which is considered as an indicator of arousal (Bradshaw, 1967; Bradley et al., 2008). It has to be noticed that, when a hard pellet had been chewed, the improvement in performance and the increase in mydriasis, observed immediately after mastication, persisted for at least 30 min. These findings could not be attributed to a learning effect, since simple test repetition did not significantly change sensorimotor performance and mydriasis, neither to other sensory properties as the hard pellet was tasteless, odorless and colorless. Moreover, it was specifically attributable to chewing and not to the motor activation in itself, since handgrip exercise induced slight but significant decreases in performance and mydriasis. Such decreases were significant only among females, likely owing to less developed musculature which may have been fatigued by the handgrip exercise, thus reducing their performance (White et al., 2013). To weigh the possible translational impact, the chewing effects have been replicated by administering a commercially available gum. The results show how the effects can be still observed, but they are short-lasting and reduced in amplitude. We argue that this might be related to the soft consistency of the commercially available pellets, as compared to the custom-made hard ones.

The improvement in cognitive performance induced by chewing could be attributed to an enhancement of the arousal level, as indicated by the task-related enhancement in mydriasis observed following the chewing period, and by the significant correlation between the changes in performance and mydriasis. Thus, these findings suggest that the sensorimotor orofacial activity has a strong impact on the modulatory system regulating arousal, likely due to the influence exerted on the ARAS and related structures by the central pattern generator for chewing and/or by the sensory signals elicited by masticatory movements. In fact, trigeminal input exerts a tonic action on the cortical activity, as indicated by the cortical synchronization induced by bilateral trigeminal neurectomy in encephale-isolé preparation (Roger et al., 1956) and by the cortical desynchronization associated with the stimulation of the trigeminal nerve in awake epileptic rats (Fanselow et al., 2000). Although the fibers arising from the trigeminal nuclei may affect several structures belonging to the ARAS (McKinley and Magoun, 1942; Semba and Fibiger, 1992; Fujise et al., 1998; Krout et al., 2002; Sakata et al., 2003), the present data suggest that, at least in part, the stimulating effects of trigeminal information can be mediated by the LC. In fact, pupil size is widely considered as a proxy of LC activity (Silvetti et al., 2013; Hoffing and Seitz, 2015; Kihara et al., 2015; Hayes and Petrov, 2016; see also Laeng et al., 2013). In the present experiments, the larger changes in task-induced mydriasis corresponded to 30% of the control value (11% of the pupil size at rest) and corresponded to 0.43 mm. In humans, changes in pupil size of 0.25–0.5 mm have been shown to correlate with the corresponding changes in the LC region activity (Alnaes et al., 2014). Within this framework, our experiments document the novel finding of a significant correlation between changes in task-induced mydriasis and performance. Trigeminal inputs to the LC have been described from the spinal and principal sensory trigeminal nuclei (Craig, 1992; Couto et al., 2006) as well as from within or near the trigeminal mesencephalic nucleus (Cedarbaum and Aghajanian, 1978; Luo et al., 1991). It has been claimed that the latter could be electrically coupled to the LC (Matsuo et al., 2015). The modulatory action of the LC on a wide range of trophic brain functions (Mather and Harley, 2016) and the degenerative effects on the brain induced by teeth loss (Kato et al., 1997; Onozuka et al., 2000; Watanabe et al., 2002; Kubo et al., 2005; Aoki et al., 2010; Oue et al., 2013) could be consistent with the speculated coupling. However, there is still no direct evidence of electrocoupling between LC neurons and trigeminal mesencephalic afferents, beyond a Fluorogold labeling passage from mesencephalic trigeminal neurons to the LC area (Fujita et al., 2012). Fibers from trigeminal nuclei could affect the distal dendrites of the pericoerulear region (Aston-Jones et al., 1986, 1991), as well as, via the paragigantocellularis and the prepositus hypoglossi nuclei (Lovick, 1986; Buisseret-Delmas et al., 1999), the LC cell bodies (Aston-Jones et al., 1986). So, an increase in LC activity could occur during the trigeminal-induced enhancements in task-related mydriasis and in cognitive performance (see De Cicco et al., 2014, 2016), which would benefit of the release of noradrenaline at cerebral cortical level (Gabay et al., 2011).

It is unknown whether trigemino-coeruleus pathways modulate the activity of noradrenergic neurons projecting to the thalamus and the cortex, which are likely implicated in the arousal. Recent evidence indicates, in fact, that the LC is heterogeneous in terms of its neurochemical composition, neuronal firing patterns, intranuclear topography of target-specific projection neurons (Uematsu et al., 2015), and sub-regional extension of peri-coeruleus dendrites (Aston-Jones et al., 1991). Further investigations are necessary in order to clarify the issue. However, cells projecting to different brain region are largely overlapping (Uematsu et al., 2015), so that trigeminal afferent input to a given LC region could potentially affect different output channels.

So, it is likely that sensorimotor trigeminal signals elicit changes in the excitability of ARAS/LC which outlast mastication for at least a few minutes and may extend up to half an hour when hard pellets are chewed.

Another relevant point emerges from the improvement in performance. In fact, this resulted from an increase in the scanning velocity of the matrices without loss in accuracy, since the increase in retrieved numbers was not associated with an increase in errors. Such an increase would have been expected on the ground of the well-known trade-off between speed and precision (Fitts, 1954; Wright and Meyer, 1983; Harris and Wolpert, 2006). These data are consistent with the widespread effect of NE release (McCormick et al., 1991), which increases the signal to noise ratio of cortical and thalamic neurons (Foote et al., 1975; Moxon et al., 2007), thus enhancing sensory coding efficiency (Fazlali et al., 2016) and, possibly, network performance in highly integrated functions (Shea-Brown et al., 2008). Finally, the observation that a higher masticatory load and, as a consequence, a stronger trigeminal input induce more significant effects on task-related mydriasis and performance with respect to a less demanding chewing task with a soft, commercially available pellet is consistent with clinical and experimental animal evidence showing how chewing soft food may lead to cognitive impairments and neurodegenerative processes (Tsutsui et al., 2007; Weijenberg et al., 2013). These data have important clinical implications, paving the way to simple behavioral strategies for preventing and/or slowing the progression of neurodegenerative disorders.

## Conclusion

Despite a few limitations (small sample size, lack of any cognitive evaluation of the participants), the present findings support the hypothesis that trigeminal activity associated with chewing activates brain arousal systems, possibly the LC, leading to persistent changes in their excitability likely improving the performance of cognitive tasks.

## Author Contributions

MPTF performed most of data analysis and gave a very important contribution to data understanding, manuscript preparation and revision. VDC contributed to the experimental design and executed the experiments. The contributions of MPTF and VDC were of the same importance for the work. MB gave an important contribution to data analysis and manuscript preparation and revision. EC, UF, and LB contributed to manuscript preparation and revision. DM contributed to the experimental design, supervised the whole activity and prepared most of the manuscript. All authors read, edited and approved the manuscript.

## Conflict of Interest Statement

The authors declare that the research was conducted in the absence of any commercial or financial relationships that could be construed as a potential conflict of interest. The reviewer KS and handling Editor declared their shared affiliation, and the handling Editor states that the process nevertheless met the standards of a fair and objective review.

## References

[B1] Allen A. P. Smith A. P. (2012). Effects of chewing gum and time-on-task on alertness and attention. *Nutr. Neurosci.* 15 176–185. 10.1179/1476830512Y.0000000009 22583804

[B2] Allen K. L. Norman R. G. Katz R. V. (2008). The effect of chewing gum on learning as measured by test performance. *Nutr. Bull.* 33 102–107. 10.1111/j.1467-3010.2008.00697.x

[B3] Alnaes D. Sneve M. H. Espeseth T. Endestad T. van de Pavert S. H. Laeng B. (2014). Pupil size signals mental effort deployed during multiple object tracking and predicts brain activity in the dorsal attention network and the locus coeruleus. *J. Vis.* 14:1 10.1167/14.4.1 24692319

[B4] Aoki H. Kimoto K. Hori N. Yamamoto Y. Onozuka M. (2010). Molarless condition suppress proliferation but not differentiation rates into neurons in the rat dentate gyrus. *Neurosci. Lett.* 469 44–48. 10.1016/j.neulet.2009.11.041 19931591

[B5] Appenteng K. Lund J. P. Seguin J. J. (1982). Intraoral mechanoreceptor activity during jaw movement in the anesthetized rabbit. *J. Neurophysiol.* 48 27–37. 7119849 10.1152/jn.1982.48.1.27

[B6] Aston-Jones G. Ennis M. Pieribone V. A. Nickell W. T. Shipley M. T. (1986). The brain nucleus locus coeruleus: restricted afferent control of a broad efferent network. *Science* 234 734–737. 10.1126/science.3775363 3775363

[B7] Aston-Jones G. Shipley M. T. Chouvet G. Ennis M. van Bockstaele E. Pieribone V. (1991). Afferent regulation of locus coeruleus neurons: anatomy, physiology and pharmacology. *Prog. Brain Res.* 88 47–75. 10.1016/S0079-6123(08)63799-1 1687622

[B8] Bradley M. M. Miccoli L. Escrig M. A. Lang P. J. (2008). The pupil as a measure of emotional arousal and autonomic activation. *Psychophysiology* 45 602–607. 10.1111/j.1469-8986.2008.00654.x 18282202 PMC3612940

[B9] Bradshaw J. (1967). Pupil size as a measure of arousal during information processing. *Nature* 216 515–516. 10.1038/216515a0 6057275

[B10] Breen L. A. Burde R. M. Loewy A. D. (1983). Brainstem connections to the Edinger-Westphal nucleus of the cat: a retrograde tracer study. *Brain Res.* 261 303–306. 10.1016/0006-8993(83)90633-9 6831211

[B11] Buisseret-Delmas C. Compoint C. Delfini C. Buisseret P. (1999). Organisation of reciprocal connections between trigeminal and vestibular nuclei in the rat. *J. Comp. Neurol.* 409 153–168. 10.1002/(SICI)1096-9861(19990621)409:1<153::AID-CNE11>3.0.CO;2-# 10363717

[B12] Carter M. E. Yizhar O. Chikahisa S. Nguyen H. Adamantidis A. Nishino S. (2010). Tuning arousal with optogenetic modulation of locus coeruleus neurons. *Nat. Neurosci.* 13 1526–1533. 10.1038/nn.2682 21037585 PMC3174240

[B13] Cedarbaum J. M. Aghajanian G. K. (1978). Afferent projections to the rat locus coeruleus as determined by a retrograde tracing technique. *J. Comp. Neurol.* 178 1–16. 10.1002/cne.901780102 632368

[B14] Clascá F. Rubio-Garrido P. Jabaudon D. (2012). Unveiling the diversity of thalamocortical neuron subtypes. *Eur. J. Neurosci.* 35 1524–1532. 10.1111/j.1460-9568.2012.08033.x 22606998

[B15] Couto L. B. Moroni C. R. dos Reis Ferreira C. M. Elias-Filho D. H. Parada C. A. Pelá I. R. (2006). Descriptive and functional neuroanatomy of locus coeruleus-noradrenaline-containing neurons involvement in bradykinin-induced antinociception on principal sensory trigeminal nucleus. *J. Chem. Neuroanat.* 32 28–45. 10.1016/j.jchemneu.2006.03.003 16678997

[B16] Craig A. D. (1992). Spinal and trigeminal lamina I input to the locus coeruleus anterogradely labeled with *Phaseolus vulgaris* leucoagglutinin (PHA-L) in the cat and the monkey. *Brain Res.* 584 325–328. 10.1016/0006-8993(92)90915-V 1515950

[B17] Davidson M. G. (2011). Herbal-caffeinated chewing gum, but not bubble gum, improves aspects of memory. *Appetite* 57 303–307. 10.1016/j.appet.2011.04.019 21570431

[B18] De Cicco V. Barresi M. Tramonti Fantozzi M. P. Cataldo E. Parisi V. Manzoni D. (2016). Oral implant-prostheses: new teeth for a brighter brain. *PLoS ONE* 11:e0148715 10.1371/journal.pone.0148715 PMC4771091 26919258

[B19] De Cicco V. Cataldo E. Barresi M. Parisi V. Manzoni D. (2014). Sensorimotor trigeminal unbalance modulates pupil size. *Arch. Ital. Biol.* 152 1–12. 25181592

[B20] Dellow P. G. Lund J. P. (1971). Evidence for central timing of rhythmical mastication. *J. Physiol.* 215 1–13. 10.1113/jphysiol.1971.sp009454 5579653 PMC1331863

[B21] Fanselow E. E. Reid A. P. Nicolelis M. A. (2000). Reduction of pentylenetetrazole-induced seizure activity in awake rats by seizure-triggered trigeminal nerve stimulation. *J. Neurosci.* 20 8160–8168. 11050139 10.1523/JNEUROSCI.20-21-08160.2000 PMC6772743

[B22] Farella M. Bakke M. Michelotti A. Martina R. (2001). Effects of prolonged gum chewing on pain and fatigue in human jaw muscles. *Eur. J. Oral Sci.* 109 81–85. 10.1034/j.1600-0722.2001.00991.x 11347660

[B23] Fazlali Z. Ranjbar-Slamloo Y. Ad M. Arabzadeh E. (2016). Correlation between cortical state and locus coeruleus activity: implications for sensory coding in rat barrel cortex. *Front. Neural Circuits* 10:14 10.3389/fncir.2016.00014 PMC4805600 27047339

[B24] Fitts P. M. (1954). The information capacity of the human motor system in controlling the amplitude of movement. *J. Exp. Psychol.* 47 381–391. 10.1037/h0055392 13174710

[B25] Foote S. L. Freedman R. Oliver A. P. (1975). Effects of putative neurotransmitters on neuronal activity in monkey auditory cortex. *Brain Res.* 86 229–242. 10.1016/0006-8993(75)90699-X 234774

[B26] Fujise T. Yoshimatsu H. Kurokawa M. Oohara A. Kang M. Nakata M. (1998). Satiation and masticatory function modulated by brain histamine in rats. *Proc. Soc. Exp. Biol. Med.* 217 228–234. 10.3181/00379727-217-44227 9452148

[B27] Fujita K. Matsuo K. Yuzuriha S. Kawagishi K. Moriizumi T. (2012). Cell bodies of the trigeminal proprioceptive neurons that transmit reflex contraction of the levator muscle are located in the mesencephalic trigeminal nucleus in rats. *J. Plast. Surg. Hand Surg.* 46 383–388. 10.3109/2000656X.2012.722094 23157498

[B28] Gabay S. Pertzov Y. Henik A. (2011). Orienting of attention, pupil size and the norepinephrine system. *Atten. Percept. Psychophys.* 73 123–129. 10.3758/s13414-010-0015-4 21258914

[B29] Gatz M. Mortimer J. A. Fratiglioni L. Johansson B. Berg S. Reynolds C. A. (2006). Potentially modifiable risk factors for dementia in identical twins. *Alzheimers Dement.* 2 110–117. 10.1016/j.jalz.2006.01.002 19595867

[B30] Harris C. M. Wolpert D. M. (2006). The main sequence of saccades optimizes speed-accuracy trade-off. *Biol. Cybern.* 95 21–29. 10.1007/s00422-006-0064-x 16555070 PMC2637438

[B31] Hayes T. R. Petrov A. A. (2016). Pupil diameter tracks the exploration-exploitation trade-off during analogical reasoning and explains individual differences in fluid intelligence. *J. Cogn. Neurosci.* 28 308–318. 10.1162/jocn_a_00895 26488587

[B32] Hess E. H. Polt J. M. (1964). Pupil size in relation to mental activity during simple problem-solving. *Science* 143 1190–1192. 10.1126/science.143.3611.1190 17833905

[B33] Hirano Y. Obata T. Kashikura K. Nonaka H. Tachibana A. Ikehira H. (2008). Effects of chewing in working memory processing. *Neurosci. Lett.* 436 189–192. 10.1016/j.neulet.2008.03.033 18403120

[B34] Hirano Y. Obata T. Takahashi H. Tachibana A. Kuroiwa D. Takahashi T. (2013). Effects of chewing on cognitive processing speed. *Brain Cogn.* 81 376–381. 10.1016/j.bandc.2012.12.002 23375117

[B35] Hirano Y. Onozuka M. (2014). Chewing and cognitive function. *Brain Nerve* 66 25–32. 24371128

[B36] Hoffing R. C. Seitz A. R. (2015). Pupillometry as a glimpse into the neurochemical basis of human memory encoding. *J. Cogn. Neurosci.* 27 765–774. 10.1162/jocn_a_00749 25390194 PMC4472441

[B37] Horio T. Kawamura Y. (1989). Effects of texture of food on chewing patterns in the human subject. *J. Oral Rehabil.* 16 177–183. 10.1111/j.1365-2842.1989.tb01331.x 2715866

[B38] Johnson A. Miles C. Haddrell B. Harrison E. Osborne L. Wilson N. (2012). The effect of chewing gum on physiological and self-rated measures of alertness and daytime sleepiness. *Physiol. Behav.* 105 815–820. 10.1016/j.physbeh.2011.10.020 22061430

[B39] Joshi S. Li Y. Kalwani R. M. Gold J. I. (2016). Relationships between pupil diameter and neuronal activity in the locus coeruleus, colliculi, and cingulate cortex. *Neuron* 89 221–234. 10.1016/j.neuron.2015.11.028 26711118 PMC4707070

[B40] Kato T. Usami T. Noda Y. Hasegawa M. Ueda M. Nabeshima T. (1997). The effect of the loss of molar teeth on spatial memory and acetylcholine release from the parietal cortex in aged rats. *Behav. Brain Res.* 83 239–242. 10.1016/S0166-4328(97)86078-0 9062693

[B41] Kihara K. Takeuchi T. Yoshimoto S. Kondo H. M. Kawahara J. I. (2015). Pupillometric evidence for the locus coeruleus-noradrenaline system facilitating attentional processing of action-triggered visual stimuli. *Front. Psychol.* 6:827 10.3389/fpsyg.2015.00827 PMC4466527 26124741

[B42] Krout K. E. Belzer R. E. Loewy A. D. (2002). Brainstem projections to midline and intralaminar thalamic nuclei of the rat. *J. Comp. Neurol.* 448 53–101. 10.1002/cne.10236 12012375

[B43] Kubo K. Iwaku F. Watanabe K. Fujita M. Onozuka M. (2005). Molarless-induced changes of spines in hippocampal region of SAMP8 mice. *Brain Res.* 1057 191–195. 10.1016/j.brainres.2005.07.038 16112090

[B44] Laeng B. Sæther L. Holmlund T. Wang C. E. Waterloo K. Eisemann M. (2013). Invisible emotional expressions influence social judgments and pupillary responses of both depressed and non-depressed individuals. *Front. Psychol.* 4:291 10.3389/fpsyg.2013.00291 PMC3660658 23734141

[B45] Lavigne G. Kim J. S. Valiquette C. Lund J. P. (1987). Evidence that periodontal pressoreceptors provide positive feedback to jaw closing muscles during mastication. *J. Neurophysiol.* 58 342–358. 3655872 10.1152/jn.1987.58.2.342

[B46] Lovick T. A. (1986). Projections from brainstem nuclei to the nucleus paragigantocellularis lateralis in the cat. *J. Auton. Nerv. Syst.* 16 1–11. 10.1016/0165-1838(86)90047-0 3009593

[B47] Lund J. P. (1991). Mastication and its control by the brain stem. *Crit. Rev. Oral Biol. Med.* 2 33–64. 10.1177/10454411910020010401 1912143

[B48] Luo P. F. Wang B. R. Peng Z. Z. Li J. S. (1991). Morphological characteristics and terminating patterns of masseteric neurons of the mesencephalic trigeminal nucleus in the rat: an intracellular horseradish peroxidase labeling study. *J. Comp. Neurol.* 303 286–299. 10.1002/cne.903030210 2013641

[B49] Mather M. Harley C. W. (2016). The locus coeruleus: essential for maintaining cognitive function and the aging brain. *Trends Cogn. Sci.* 20 214–226. 10.1016/j.tics.2016.01.001 26895736 PMC4761411

[B50] Matsuo K. Ban R. Hama Y. Yuzuriha S. (2015). Eyelid opening with trigeminal proprioceptive activation regulates a brainstem arousal mechanism. *PLoS ONE* 10:e0134659 10.1371/journal.pone.0134659 PMC4526522 26244675

[B51] Matsutani K. Tsuruoka M. Shinya A. Furuya R. Kawawa T. (2000). Stimulation of the locus coeruleus suppresses trigeminal sensorimotor function in the rat. *Brain Res. Bull.* 53 827–832. 10.1016/S0361-9230(00)00426-3 11179850

[B52] McCormick D. A. Pape H. C. Williamson A. (1991). Actions of norepinephrine in the cerebral cortex and thalamus: implications for function of the central noradrenergic system. *Prog. Brain Res.* 88 293–305. 10.1016/S0079-6123(08)63817-0 1726028

[B53] McKinley W. A. Magoun H. W. (1942). The bulbar projection of the trigeminal nerve. *Am. J. Physiol.* 137 217–224.

[B54] Moxon K. A. Devilbiss D. M. Chapin J. K. Waterhouse B. D. (2007). Influence of norepinephrine on somatosensory neuronal responses in the rat thalamus: a combined modeling and in vivo multi-channel, multi-neuron recording study. *Brain Res.* 1147 105–123. 10.1016/j.brainres.2007.02.006 17368434 PMC4529675

[B55] Murphy P. R. O’Connell R. G. O’Sullivan M. Robertson I. H. Balsters J. H. (2014). Pupil diameter covaries with BOLD activity in human locus coeruleus. *Hum. Brain Mapp.* 35 4140–4154. 10.1002/hbm.22466 24510607 PMC6869043

[B56] Ohkubo C. Morokuma M. Yoneyama Y. Matsuda R. Lee J. S. (2013). Interactions between occlusion and human brain function activities. *J. Oral Rehabil.* 40 119–129. 10.1111/j.1365-2842.2012.02316.x 22624951

[B57] Okamoto N. Morikawa M. Okamoto K. Habu N. Iwamoto J. Tomioka K. (2010). Relationship of tooth loss to mild memory impairment and cognitive impairment: findings from the Fujiwara-kyo study. *Behav. Brain Funct.* 6:77 10.1186/1744-9081-6-77 PMC3024962 21194415

[B58] Onozuka M. Watanabe K. Nagasaki S. Ozono S. Nishiyama K. Kawase T. (2000). Impairment of spatial memory and changes in astroglial responsiveness following loss of molar teeth in aged SAMP8 mice. *Behav. Brain Res.* 108 145–155. 10.1016/S0166-4328(99)00145-X 10701658

[B59] Onyper S. V. Carr T. L. Farrar J. S. Floyd B. R. (2011). Cognitive advantages of chewing gum. Now you see them, now you don’t. *Appetite* 57 321–328. 10.1016/j.appet.2011.05.313 21645566

[B60] Oue H. Miyamoto Y. Okada S. Koretake K. Jung C. G. Michikawa M. (2013). Tooth loss induces memory impairment and neuronal cell loss in APP transgenic mice. *Behav. Brain Res.* 252 318–325. 10.1016/j.bbr.2013.06.015 23773908

[B61] Peyron M. A. Lassauzay C. Woda A. (2002). Effects of increased hardness on jaw movement and muscle activity during chewing of visco-elastic model foods. *Exp. Brain Res.* 142 41–51. 10.1007/s00221-001-0916-5 11797083

[B62] Rajkowski J. Kubiak P. Aston-Jones G. (1993). Correlations between locus coeruleus (LC) neural activity, pupil diameter and behaviour in monkey support a role of LC in attention. *Proc. Soc. Neurosci. Abstr.* 19 974.

[B63] Rajkowski J. Kubiak P. Aston-Jones G. (1994). Locus coeruleus activity in monkey: phasic and tonic changes are associated with altered vigilance. *Brain Res. Bull.* 35 607–616. 10.1016/0361-9230(94)90175-9 7859118

[B64] Roger A. Rossi G. F. Zirondoli A. (1956). Le rôle des afférences des nerfs crâniens dans le maintien de l’état vigile de la préparation “encéphale isolé”. *Electroencephalogr. Clin. Neurophysiol.* 8 1–13. 10.1016/0013-4694(56)90027-x 13294059

[B65] Sakamoto K. Nakata H. Kakigi R. (2009). The effect of mastication on human cognitive processing: a study using event-related potentials. *Clin. Neurophysiol.* 120 41–50. 10.1016/j.clinph.2008.10.001 19026594

[B66] Sakata T. Yoshimatsu H. Masaki T. Tsuda K. (2003). Anti-obesity actions of mastication driven by histamine neurons in rats. *Exp. Biol. Med.* 228 1106–1110. 10.1177/153537020322801002 14610247

[B67] Samuels E. R. Szabadi E. (2008). Functional neuroanatomy of the noradrenergic locus coeruleus: its roles in the regulation of arousal and autonomic function part I: principles of functional organisation. *Curr. Neuropharmacol.* 6 235–253. 10.2174/157015908785777229 19506723 PMC2687936

[B68] Semba K. Fibiger H. C. (1992). Afferent connections of the laterodorsal and the pedunculopontine tegmental nuclei in the rat: a retro- and antero-grade transport and immunohistochemical study. *J. Comp. Neurol.* 323 387–410. 10.1002/cne.903230307 1281170

[B69] Shea-Brown E. Gilzenrat M. S. Cohen J. D. (2008). Optimization of decision making in multilayer networks: the role of locus coeruleus. *Neural Comput.* 20 2863–2894. 10.1162/neco.2008.03-07-487 18624653

[B70] Silvetti M. Seurinck R. van Bochove M. E. Verguts T. (2013). The influence of the noradrenergic system on optimal control of neural plasticity. *Front. Behav. Neurosci.* 7:160 10.3389/fnbeh.2013.00160 PMC3826478 24312028

[B71] Smith A. (2009). Effects of chewing gum on mood, learning, memory and performance of an intelligence test. *Nutr. Neurosci.* 12 81–89. 10.1179/147683009X423247 19356310

[B72] Spinnler H. Tognoni G. (1987). Italian standardization and classification of neuropsychological tests. The Italian group on the neuropsychological study of aging. *Ital. J. Neurol. Sci.* 6(Suppl. 8), 1–120. 3330072

[B73] Szabadi E. Bradshaw C. (1996). Autonomic pharmacology ofα2-adrenoceptors. *J. Physicopharmacol.* 10 6–18.

[B74] Tsutsui K. Kaku M. Motokawa M. Tohma Y. Kawata T. Fujita T. (2007). Influences of reduced masticatory sensory input from soft-diet feeding upon spatial memory/learning ability in mice. *Biomed. Res.* 28 1–7. 10.2220/biomedres.28.1 17379951

[B75] Tucha O. Mecklinger L. Maier K. Lange K. W. (2004). Chewing gum differentially affects aspects of attention in healthy subjects. *Appetite* 42 327–329. 10.1016/j.appet.2004.01.003 15183924

[B76] Uematsu A. Tan B. Z. Johansen J. P. (2015). Projections specificity in heterogeneous locus coeruleus cell populations: implications for learning and memory. *Learn. Mem.* 22 444–451. 10.1101/lm.037283.114 26330494 PMC4561410

[B77] Wang N. May P. J. (2008). Peripheral muscle targets and central projections of the mesencephalic trigeminal nucleus in macaque monkeys. *Anat. Rec.* 291 974–987. 10.1002/ar.20712 PMC2859174 18461596

[B78] Watanabe K. Ozono S. Nishiyama K. Saito S. Tonosaki K. Fujita M. (2002). The molarless condition in aged SAMP8 mice attenuates hippocampal Fos induction linked to water maze performance. *Behav. Brain Res.* 128 19–25. 10.1016/S0166-4328(01)00268-6 11755686

[B79] Weijenberg R. A. Lobbezoo F. Knol D. L. Tomassen J. Scherder E. J. (2013). Increased masticatory activity and quality of life in elderly persons with dementia–a longitudinal matched cluster randomized single-blind multicenter intervention study. *BMC Neurol.* 13:26 10.1186/1471-2377-13-26 PMC3615955 23496988

[B80] White C. Dixon K. Samuel D. Stokes M. (2013). Handgrip and quadriceps muscle endurance testing in young adults. *Springerplus* 2:451 10.1186/2193-1801-2-451 PMC3776088 24052933

[B81] Wilhelm B. Giedke H. Lüdtke H. Bitter E. Hofmann A. Wilheim H. (2011). Daytime variations in central nervous system activation measured by a pupillographic sleepness test. *J. Sleep Res.* 10 1–7. 10.1046/j.1365-2869.2001.00239.x 11285049

[B82] Wright C. E. Meyer D. E. (1983). Conditions for a linear speed–accuracy trade-off in aimed movements. *Q. J. Exp. Psychol. A* 35 279–296. 10.1080/14640748308402134 6571311

