# Supplementary material for: Short-Term Effects of Chewing on Task Performance and Task-Induced Mydriasis: Trigeminal Influence on the Arousal Systems
Source: Front Neuroanat. 2017 Aug 8;11:68. doi: 10.3389/fnana.2017.00068 (PMC5550729; doi:10.3389/fnana.2017.00068)
Supplement: Supplementary file 1 [file Table_1.DOCX]

| **VARIABLE** | **EFFECT** | **P** | **η^2^** | **POST-HOC (T-Test)** | **P** | |
| --- | --- | --- | --- | --- | --- | --- |
| **Performance Index** | Condition  F(3,81)=23.94 | 0.0005 | 0.47 | No Activity>Handgrip  No Activity<Hard Pellet  No Activity<Soft Pellet  Handgrip<Hard Pellet  Handgrip<Soft Pellet  Hard Pellet>Soft Pellet | 0.001  0.0005  0.0005  0.0005  0.0005  0.0005 | |
|  | Time  F(2,54)=5.38 | 0.015 | 0.17 | T0<T7  T0<T37  T7>T37 | 0.0005  0.0005  0.0005 | |
|  | Condition x Time F(6,162)=9.48 | 0.0005 | 0.26 | Decomposed in Table 2 | | |
| **Scanning Velocity** | Condition  F(3,81)=4.47 | 0.006 | 0.14 | No Activity>Handgrip  No Activity<Hard Pellet  No Activity<Soft Pellet  Handgrip<Hard Pellet  Handgrip<Soft Pellet  Hard Pellet>Soft Pellet | | 0.035  0.0005  0.0005  0.0005  0.0005  0.0005 |
|  | Condition x Time  F(6,162)=3.76 | 0.002 | 0.12 | Decomposed in Table 2 | | |
| **Error**  **Rate** | Time  F(2,54)=4.42 | 0.017 | 0.14 | T0<T7  T0<T37  T7-T37 | | 0.0005  0.0005  NS |

Table 1. Statistical significant effects and interactions observed for performance parameters relative to the Spinnler-Tognoni matrices test
