# Supplementary material for: Short-Term Effects of Chewing on Task Performance and Task-Induced Mydriasis: Trigeminal Influence on the Arousal Systems
Source: Front Neuroanat. 2017 Aug 8;11:68. doi: 10.3389/fnana.2017.00068 (PMC5550729; doi:10.3389/fnana.2017.00068)
Supplement: Supplementary file 2 [file Table_2.DOCX]

|  | | **VARIABLE** | | | | | | | | |
| --- | --- | --- | --- | --- | --- | --- | --- | --- | --- | --- |
|  | | **Performance Index** | | | **Scanning Velocity** | | | **Error Rate** | | |
|  |  | **T0** | **T7** | **T37** | **T0** | **T7** | **T37** | **T0** | **T7** | **T37** |
|  | **No Activity** | 1.72±0.52 | 1.76±0.54 | 1.76±0.54 | 12.94±2.22 | 12.84±2.30 | 12.74±2.22 | 0.32±0.30 | 0.22±0.22 | 0.22±0.22 |
|  | **Post-Hoc** | **T0-T7** NS | **T7-T37** NS | **T0-T37** NS | **T0-T7** NS | **T7-T37** NS | **T0-T37** NS | **T0-T7** P<0.0005 | **T7-T37** NS | **T0-T37** P<0.0005 |
|  | **Handgrip** | 1.74±0.54 | 1.66±0.46 | 1.72±0.48 | 12.84±2.18 | 12.42±1.92 | 12.66±1.96 | 0.28±0.20 | 0.22±0.14 | 0.22±0.18 |
|  | **Post-Hoc** | **T0-T7** P<0.01 | **T7-T37** P<0.004 | **T0-T37** NS | **T0-T7** P<0.003 | **T7-T37** P<0.043 | **T0-T37** NS | **T0-T7** P<0.014 | **T7-T37** NS | **T0-T37** P<0.004 |
|  | **Hard Pellet** | 1.78± 0.50 | 2.40± 0.58 | 2.24±0.54 | 13.06±1.90 | 15.34±1.90 | 14.80±1.80 | 0.26±0.20 | 0.22±0.12 | 0.22±0.18 |
|  | **Post-Hoc** | **T0-T7** P<0.0005 | **T7-T37** P<0.001 | **T0-T37** P<0.0005 | **T0-T7** P<0.0005 | **T7-T37** P<0.004 | **T0-T37** P<0.0005 | **T0-T7** NS | **T7-T37** NS | **T0-T37** NS |
|  | **Soft Pellet** | 1.76±0.54 | 2.08±0.58 | 1.86±0.54 | 13.02±2.04 | 14.02±2.22 | 13.08±2.18 | 0.26±0.20 | 0.18±0.16 | 0.18±0.18 |
|  | **Post-Hoc** | **T0-T7** P<0.0005 | **T7-T37** P<0.0005 | **T0-T37** P<0.0005 | **T0-T7** P<0.0005 | **T7-T37** P<0.0005 | **T0-T37** NS | **T0-T7** P<0.001 | **T7-T37** NS | **T0-T37** P<0.0005 |

**CONDITION**

Table 2. Average±SD values of the different performance parameters obtained in the 4 conditions analysed at times 0 (T0), 7 (T7) and 37 (T37) minutes from the beginning of the session, i.e. before the activity/no activity period, immediately and 30 minutes after its end, respectively. All data are expressed in numbers/sec.
