# Supplementary material for: Short-Term Effects of Chewing on Task Performance and Task-Induced Mydriasis: Trigeminal Influence on the Arousal Systems
Source: Front Neuroanat. 2017 Aug 8;11:68. doi: 10.3389/fnana.2017.00068 (PMC5550729; doi:10.3389/fnana.2017.00068)
Supplement: Supplementary file 3 [file Table_3.DOCX]

|  | | **VARIABLE** | | | | | | | | | | | |
| --- | --- | --- | --- | --- | --- | --- | --- | --- | --- | --- | --- | --- | --- |
|  | | **Retrieved Numbers** | | | **Wrongly Underlined Numbers** | | | **Missed Numbers** | | | **Accuracy Percentage** | | |
|  |  | **T0** | **T7** | **T37** | **T0** | **T7** | **T37** | **T0** | **T7** | **T37** | **T0** | **T7** | **T37** |
|  | **No Activity** | 25.70±7.74 | 26.37±8.03 | 26.33±8.11 | 4.33±3.92 | 3.07±2.66 | 3.03±3.03 | 0.50±0.90 | 0.40±0.81 | 0.37±0.61 | 98% | 99% | 99% |
|  | **Hand Grip** | 25.97±8,07 | 24.90±6.92 | 25.83±7.21 | 3.63±2.55 | 2.83±1.62 | 2.83±2.35 | 0.53±0.68 | 0.50±0.78 | 0.47±0.78 | 98% | 98% | 98% |
|  | **Hard Pellet** | 26.70±7.39 | 35.97±8.83 | 33.73±8.04 | 3.57±2.64 | 3.07±1.51 | 3.23±2.46 | 0.27±0.58 | 0.30±0.65 | 0.37±0.72 | 99% | 99% | 99% |
|  | **Soft Pellet** | 26.40±7.96 | 31.07±8.80 | 27.80±8.17 | 3.60±2.47 | 2.47±2.19 | 2.53±2.34 | 0.37±0.72 | 0.20±0.55 | 0.27±0.58 | 99% | 99% | 99% |

Table 3. Average±SD values observed at the different times and in the different conditions for correctly retrieved, wrongly underlined and missed items. Accuracy percentage corresponds to the ratio of retrieve/(retrieved+missed) items.

**CONDITION**
