# Supplementary material for: Short-Term Effects of Chewing on Task Performance and Task-Induced Mydriasis: Trigeminal Influence on the Arousal Systems
Source: Front Neuroanat. 2017 Aug 8;11:68. doi: 10.3389/fnana.2017.00068 (PMC5550729; doi:10.3389/fnana.2017.00068)
Supplement: Supplementary file 4 [file Table_4.DOCX]

| **Variable** | **Effect** | **P** | **η^2^** | **Post-Hoc (T-Test)** | **P** |
| --- | --- | --- | --- | --- | --- |
| **Pupil Size (Task)** | Condition  F(3,81)=15.70 | 0.0005 | 0.37 | No Activity>Handgrip  No Activity<Hard Pellet  No Activity-Soft Pellet  Handgrip<Hard Pellet  Handgrip<Soft Pellet  Hard Pellet>Soft Pellet | 0.0005  0.0005  NS  0.0005  0.0005  0.0005 |
|  | Condition x Time  F(6,162)=8.63 | 0.0005 | 0.24 | Decomposed in Table 4 | |
| **Mydriasis** | Condition  F(3,81)=13.32 | 0.0005 | 0.33 | No Activity>Handgrip  No Activity<Hard Pellet  No Activity/Soft Pellet  Handgrip<Hard Pellet  Handgrip<Soft Pellet  Hard Pellet>Soft Pellet | 0.0005  0.0005  NS  0.0005  0.0005  0.0005 |
|  | Condition x Time  F(6,162)=7.14 | 0.0005 | 0.21 | Decomposed in Table 4 | |
|  | Gender  F(1,27)=6.11 | 0.020 | 0.19 | Males>Females | 0.020 |

Table 4. Statistical significant effects and interactions observed for pupil size during task and for task-related mydriasis.
