# Supplementary material for: Short-Term Effects of Chewing on Task Performance and Task-Induced Mydriasis: Trigeminal Influence on the Arousal Systems
Source: Front Neuroanat. 2017 Aug 8;11:68. doi: 10.3389/fnana.2017.00068 (PMC5550729; doi:10.3389/fnana.2017.00068)
Supplement: Supplementary file 5 [file Table_5.DOCX]

|  |  | **VARIABLE** | | | | | | | | |
| --- | --- | --- | --- | --- | --- | --- | --- | --- | --- | --- |
|  |  | **Pupil Size (Rest)** | | | **Pupil Size (Task)** | | | **Mydriasis** | | |
|  |  | **T0** | **T7** | **T37** | **T0** | **T7** | **T37** | **T0** | **T7** | **T37** |
|  | **No Activity** | 3.88±0.76 | 3.88±0.75 | 3.90±0.76 | 5.34±0.92 | 5.33±0.90 | 5.31±0.89 | 1.46±0.34 | 1.45±0.31 | 1.41±0.32 |
|  | **Post-Hoc** |  |  |  | **T0-T7** NS | **T7-T37** NS | **T0-T37** NS | **T0-T7** NS | **T7-T37** NS | **T0-T37**  NS |
|  | **Handgrip** | 3.89±0.76 | 3.92±0.79 | 3.85±0.74 | 5.25±0.92 | 5.04±0.86 | 5.13±0.88 | 1.36 ± 0.39 | 1.12 ± 0.37 | 1.28 ± 0.34 |
|  | **Post-Hoc** |  |  |  | **T0-T7** P<0.0005 | **T7-T37** P<0.009 | **T0-T37** P<0.003 | **T0-T7** P<0.0005 | **T7-T37** P<0.0005 | **T0-T37** P<0.041 |
|  | **Hard Pellet** | 3.83±0.74 | 3.84±0.73 | 3.82±0.74 | 5.27±0.92 | 5.69±0.98 | 5.57±0.95 | 1.43±0.36 | 1.86±0.41 | 1.75±0.34 |
|  | **Post-Hoc** |  |  |  | **T0-T7** P<0.0005 | **T7-T37** P<0.003 | **T0-T37**  P<0.0005 | **T0-T7** P<0.0005 | **T7-T37** P<0.003 | **T0-T37** P<0.0005 |
|  | **Soft Pellet** | 3.84±0.77 | 3.88±0.72 | 3.88±0.73 | 5.28±0.93 | 5.51±0.90 | 5.28±0.92 | 1.44±0.35 | 1.63±0.40 | 1.40±0.35 |
|  | **Post-Hoc** |  |  |  | **T0-T7** P<0.0005 | **T7-T37** P<0.0005 | **T0-T37** NS | **T0-T7** P<0.0005 | **T7-T37** P<0.0005 | **T0-T37** NS |

**CONDITION**

Table 5. Average±SD values of the different pupil size parameters obtained in the 4 conditions analysed at times 0 (T0), 7 (T7) and 37 (T37) minutes from the beginning of the session, i.e. before the activity/no activity period, immediately and 30 minutes after its end, respectively. All data are expressed in mm.
